# Risk of earlier atherosclerotic cardiovascular disease in women with low bone mineral density

**DOI:** 10.1038/s41598-022-19801-5

**Published:** 2022-09-26

**Authors:** Jiesuck Park, Kyoung Min Kim, Yeonyee E. Yoon, In-Chang Hwang, Goo-Yeong Cho

**Affiliations:** 1 Department of Cardiology, Cardiovascular Center, Seoul National University Bundang Hospital, Seongnam, Gyeonggi-do Republic of Korea; 2 Department of Internal Medicine, Seoul National University College of Medicine, Seoul, Republic of Korea; 3 Division of Endocrinology, Department of Internal Medicine, Yongin Severance Hospital, Yonsei University College of Medicine, Yongin, Gyeonggi-do Republic of Korea

**Keywords:** Cardiology, Endocrinology

## Abstract

Low bone mineral density (BMD) is associated with higher risk of atherosclerotic cardiovascular disease (ASCVD) in women. We investigated whether the association between low BMD and ASCVD differs according to the age at ASCVD occurrence. We retrospectively analyzed 7932 women aged 50–65 years who underwent dual-energy X-ray absorptiometry. ASCVD was defined as a composite of ASCVD death, myocardial infarction, and ischemic stroke. When we classified participants into no event (n = 7803), early ASCVD (< 70 years) (n = 97), and late ASCVD (≥ 70 years) (n = 32) groups, age gradually increased across groups (median, 58, 60, and 63 years, respectively). However, the estimated BMD T-score at the age of 65 years was lowest in the early ASCVD group (median − 0.9, − 1.1, and − 0.5, respectively). Lower BMD was an independent predictor for early ASCVD (adjusted hazard ratio [95% confidence interval]: 1.34 [1.08–1.67] per 1-SD decrease in T-score), but not for late ASCVD (0.88 [0.60–1.30]). The inverse trend between early ASCVD risk and BMD T-score was consistent regardless of the number of accompanied clinical risk factors. Thus, low BMD is an independent predictor for early ASCVD in women. BMD evaluation can provide prognostic benefit for risk stratification for early ASCVD.

## Introduction

Osteoporosis and atherosclerosis are both representative age-related diseases that are implicated in a significant rise in morbidity and mortality globally^1^. Diverse clinical risk factors contribute individually to the occurrence and progression of these diseases during aging, including familial histories, menopausal status, low body weight, etc. for osteoporosis, and dyslipidemia, diabetes, and hypertension (HTN), etc. for atherosclerosis^2^. Furthermore, epidemiologic studies indicate an increased risk of atherosclerotic cardiovascular disease (ASCVD) in individuals with low bone mineral density (BMD) and vice versa^1,3–5^. Additionally, in our recent longitudinal cohort study, we found a significant association between low BMD and ASCVD risk, independent of age and other conventional clinical risk factors^6^. Thus, although the mechanisms that underlie the association between osteoporosis and atherosclerosis remain unclear, accumulating evidence indicates potential links between these two conditions via aging-dependent or -independent pathways.

The incidence of ASCVD increases during the aging process, along with a growing prevalence of cardiovascular risk factors^7^. While age is the most vital and non-modifiable risk factor for ASCVD, coexisting risk factors play an important role in the acceleration of the disease course and might lead an earlier presentation of ASCVD, beyond the risk related to aging per se^8,9^. However, to date, studies on whether the prognostic impact of low BMD for the development of ASCVD may differ according to the age of ASCVD onset are lacking. A differential predictive value of low BMD for an earlier or later occurrence of ASCVD would provide more clues regarding the relationship between osteoporosis and ASCVD. Therefore, in the present study, we investigated whether the association between low BMD and ASCVD differs according to the age of ASCVD occurrence.

## Methods

### Study population

**Figure 1:**
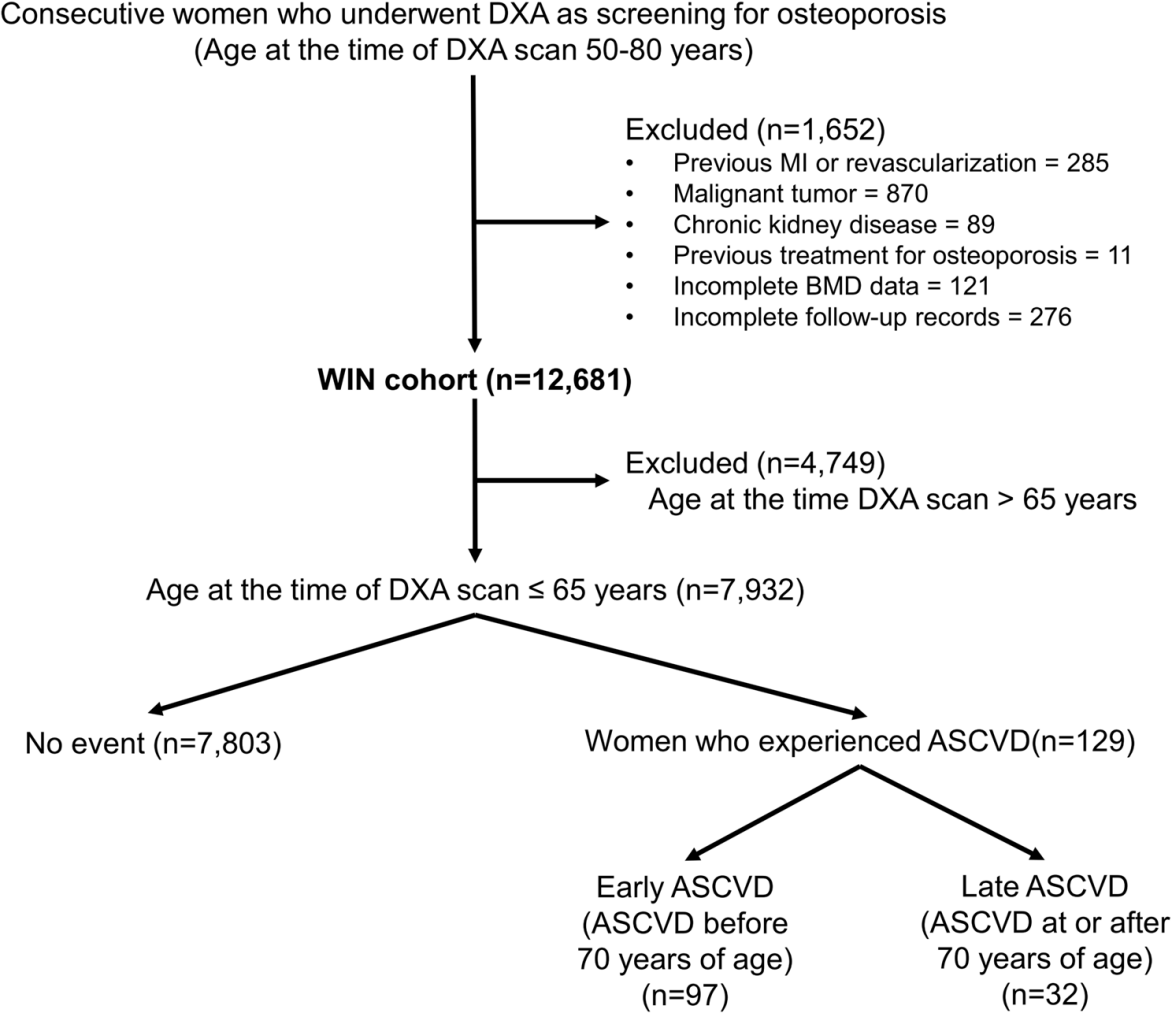
Study flow. From the Women Study for the Interaction between Bone and Vascular Health (WIN cohort), a total of 7932 women aged 65 years or lower who underwent DXA as osteoporosis screening were analyzed. Participants were categorized according to the outcome event, where 129 women experienced ASCVD. Women with ASCVD were further stratified based on the age at the event as early (< 70 years) and late ASCVD (≥ 70 years). *ASCVD* atherosclerotic cardiovascular disease, *BMD* bone mineral density, *DXA* dual-energy X-ray absorptiometry, *MI* myocardial infarction.

Study participants were recruited from the Women Study for the Interaction between Bone and Vascular Health (WIN cohort), which enrolled 12,681 women aged 50–80 years who underwent dual-energy X-ray absorptiometry (DXA) for osteoporosis screening at Seoul National University Bundang Hospital between 2005 and 2014^6^. The exclusion criteria were a history of myocardial infarction (MI) or coronary revascularization, malignancy, chronic kidney disease, previous osteoporosis treatment, incomplete BMD measurement data, and incomplete follow-up data (Fig. 1). None of the subjects had received hormone replacement therapy for more than 180 days within six months before and after the index DXA examination. In the current study, we limited the study population to those aged 65 years or younger at the time of the DXA scan, to investigate the differential impact of low BMD on earlier and later ASCVD occurrence and secure an adequate follow-up duration. After applying the study criteria, a total of 7932 women remained for analysis (Fig. 1). The study protocol complied with the principles of the Declaration of Helsinki and was approved by the institutional review board of Seoul National University Bundang Hospital (IRB No. B1806-474-001). The board waived the requirement for informed consent because of the retrospective study design.

### Data collection and definition of clinical risk factors

Clinical data were obtained by a dedicated review of the electronic medical records at the study institution, which was founded in fully digitalized form^10^. We included age, body mass index (BMI), HTN, type 2 diabetes mellitus (T2DM), hyperlipidemia (HL), current smoking, and previous fracture as conventional clinical risk factors. The diagnosis of risk factors was defined as previously validated^11,12^: HTN was defined as the ascertainment of a diagnosis of HTN, blood pressure ≥ 140/90 mmHg, or the administration of an anti-hypertensive drug within 6 months of the index DXA scan; T2DM was defined as the ascertainment of a diagnosis, fasting plasma glucose level ≥ 126 mg/dL, hemoglobin A1c level ≥ 6.5%, or the administration of an anti-diabetic drug within 6 months of the index DXA scan; and HL was defined as the ascertainment of a diagnosis of HL, total cholesterol level ≥ 240 mg/dL, low-density lipoprotein cholesterol level ≥ 160 mg/dL, or treatment with statins within 6 months of the index DXA scan. BMI was calculated by the weight (kg) divided by the square of the height (m^2^) recorded at the initial DXA scan.

### BMD measurement and diagnosis for osteoporosis

We used a single DXA scanner (Lunar Prodigy; GE, Madison, Wisconsin, USA) to measure the BMD at the lumbar spine, femoral neck, and total hip area, following the manufacturer’s protocol^13^. A previous study demonstrated that the total hip BMD was the most relevant value for ASCVD risk^6^; thus, we used the total hip BMD as a reference value of the BMD profile. The T-score was calculated using the standard reference for Asian populations provided by the manufacturer. Osteopenia was defined as a BMD T-score between − 1.0 and − 2.5, and osteoporosis was defined as a T-score below − 2.5, in accordance with World Health Organization guidelines^14^. The precision error (percent coefficient of variation) was < 2% for the BMD measurement.

### Definition of study outcomes

ASCVD was the primary outcome, defined as a composite endpoint of ASCVD death, non-fatal MI, and non-fatal ischemic stroke^6^. For ASCVD death, the date and cause of death were obtained from the Cause of Death Statistics from Statistics Korea. Event data for non-fatal MI and ischemic stroke were acquired through a dedicated review of electronic medical records, performed by an independent investigator blinded to the baseline clinical characteristics and BMD. Participants were followed up from the date of the index DXA scan, and were censored at the first event of any component of the primary outcome or the last date of follow-up for those without an event or lost to follow-up. To investigate the differential impact of low BMD on earlier and later ASCVD occurrence, we further stratified study participants according to the age at the time of ASCVD; those who experienced ASCVD before the age of 70 years were classified into the early ASCVD group, and those who experienced ASCVD at or after the age of 70 years were classified into the late ASCVD group^7^.

### Statistical analysis

Clinical data are described as the median with interquartile range (IQR) for continuous variables, and as the number with percentage for factorial variables. Baseline clinical features, including the BMD T-score, of early and late ASCVD groups were compared to those of the no event group, with significant differences examined using the Kruskal–Wallis test and Chi-square test, as appropriate. Since the age at the index DXA scan differed among participants, we additionally estimated the total hip BMD T-score at the age of 65 years. Specifically, after building a linear regression model with age and BMD T-score for the entire study population, the model coefficient was derived to estimate the decrease in BMD T-score per 1-year increase in age. Based on this estimation, the BMD T-score at the age of 65 years was calculated for each participant. The independent association of clinical risk factors (age, BMI, HTN, T2DM, HL, current smoking, and previous fracture) and the BMD T-score with early and late ASCVD was evaluated using the multivariable-adjusted Cox regression hazard model. Estimated hazard ratios (HRs) of the predictors for early and late ASCVD were compared to evaluate differences between early and late ASCVD in the association of the predictors. The proportional hazard assumption was checked using the Schoenfeld residual test, which confirmed no violations for the prediction models for overall, early, and late ASCVD.

We further evaluated whether low BMD could improve risk stratification for early ASCVD over conventional clinical risk factors. The predicted risk of early ASCVD was estimated using the final hazard model including clinical risk factors and the BMD T-score. The trend of the predicted risk of early ASCVD was then plotted across the range of T-scores and number of clinical risk factors.

All statistical analyses were performed using R (version 4.1.1; R Development Core Team, Vienna, Austria. URL https://www.R-project.org/). We used *survival* (version 3.1-12) package for plotting cumulative incidence curves, multivariate analysis for independent predictors, and estimation for the predicted risk of early ASCVD. Two-sided p-values < 0.05 were considered statistically significant.

## Results

### Baseline clinical features and BMD profile

Clinical characteristics of the 7932 women (age 58 [54–62] years; BMI 23.6 [21.8–25.6] kg/m^2^; BMD T-score: − 0.4 [− 1.0 to 0.3]) are summarized in Supplementary Table S1. During follow-up (9.8 [3.8–11.8] years), 129 women experienced ASCVD; 97 experienced early ASCVD (at < 70 years of age) and 32 experienced late ASCVD (at ≥ 70 years of age) (Fig. 1). When stratified by the occurrence of ASCVD (Supplementary Table S1), women who developed ASCVD were older (58 [54–62] vs. 61 [58–63] years; p < 0.001) and had higher prevalences of T2DM (8.2% vs. 32.6%; p < 0.001), HL (35.8% vs. 60.5%; p < 0.001), and current smoking (1.1% vs. 5.4%; p < 0.001) than women in the no event group. Furthermore, women who developed ASCVD showed a significantly lower BMD T-score (− 0.6 [− 1.2 to 0.1] vs. − 0.4 [− 1.0 to 0.3]; p = 0.009), and significantly greater tendency to be diagnosed with osteopenia and osteoporosis (p < 0.001), than women in the no event group.

When women with ASCVD were further stratified according to the age at which ASCVD occurred, age and HL prevalence gradually increased across the no event, early ASCVD, and late ASCVD groups (Table 1). The prevalences of other clinical risk factors were comparable between early and late ASCVD groups. The baseline BMD T-score was significantly lower in the early ASCVD group than in the no event group (− 0.7 [− 1.3 to 0.1] vs. − 0.4 [− 1.0 to 0.3]; p = 0.005), but did not differ between the late ASCVD and no event groups (− 0.5 [− 0.8 to 0.0] vs. − 0.4 [− 1.0 to 0.3]; p = 0.728). Similarly, the prevalences of osteopenia and osteoporosis were significantly higher in the early ASCVD group than in the no event group (p < 0.001), but did not differ between the late ASCVD and no event groups (p = 0.516) (Table 1). The estimated BMD T-score at the age of 65 years was significantly lower in the early ASCVD group (− 1.1 [− 1.7 to − 0.3]) than in the no event (− 0.9 [− 1.5 to − 0.2]; p = 0.037) and late ASCVD groups (− 0.5 [− 1.0 to 0.0]; p = 0.008), but did not differ between the late ASCVD and no event groups (p = 0.056) (Supplementary Fig. S1).

**Table 1:** Baseline characteristics.

	No event (n = 7803)	Early ASCVD (n = 97)	p-value vs. no event	Late ASCVD (n = 32)	p-value vs. no event	p-value early vs. late
Age, years	58 (54–62)	60 (57–63)	0.006	63 (62–65)	< 0.001	< 0.001
Follow-up duration, years	9.8 (3.8–11.8)	4.9 (2.3–8.0)	< 0.001	9.5 (8.3–10.4)	0.836	< 0.001
BMI, kg/m^2^	23.6 (21.8–25.6)	24.0 (22.1–25.7)	0.343	24.2 (22.4–25.9)	0.417	0.735
Hypertension	2,345 (30.1)	28 (28.9)	0.887	10 (31.2)	0.999	0.974
Type 2 diabetes	642 (8.2)	30 (30.9)	< 0.001	12 (37.5)	< 0.001	0.638
Hyperlipidemia	2,793 (35.8)	52 (53.6)	< 0.001	26 (81.2)	< 0.001	0.010
Current smoking	87 (1.1)	6 (6.2)	< 0.001	1 (3.1)	0.813	0.832
Previous fracture	177 (2.3)	4 (4.1)	0.383	1 (3.1)	0.999	0.999
Total hip BMD	0.890 (0.811–0.974)	0.852 (0.777–0.942)	0.007	0.875 (0.839–0.932)	0.533	0.341
Total hip BMD T-score	− 0.4 (− 1.0 to 0.3)	− 0.7 (− 1.3 to 0.1)	0.005	− 0.5 (− 0.8 to 0.0)	0.728	0.197
**Diagnosis of osteopenia and osteoporosis based on total hip BMD T-score**						
Normal BMD (− 1 < T-score)	5,642 (72.3)	58 (59.8)	< 0.001	25 (78.1)	0.516	0.168
Osteopenia (− 2.5 < T-score ≤ − 1)	2,040 (26.1)	32 (33.0)		6 (18.8)		
Osteoporosis (T-score ≤ − 2.5)	121 (1.6)	7 (7.2)		1 (3.1)		

Data are presented as the median (interquartile range) for continuous variables and number (percentage) for categorical variables.

*ASCVD* atherosclerotic cardiovascular disease, *BMD* bone mineral density, *BMI* body mass index.

### Comparisons in the independent predictors for early and late ASCVD

**Figure 2:**
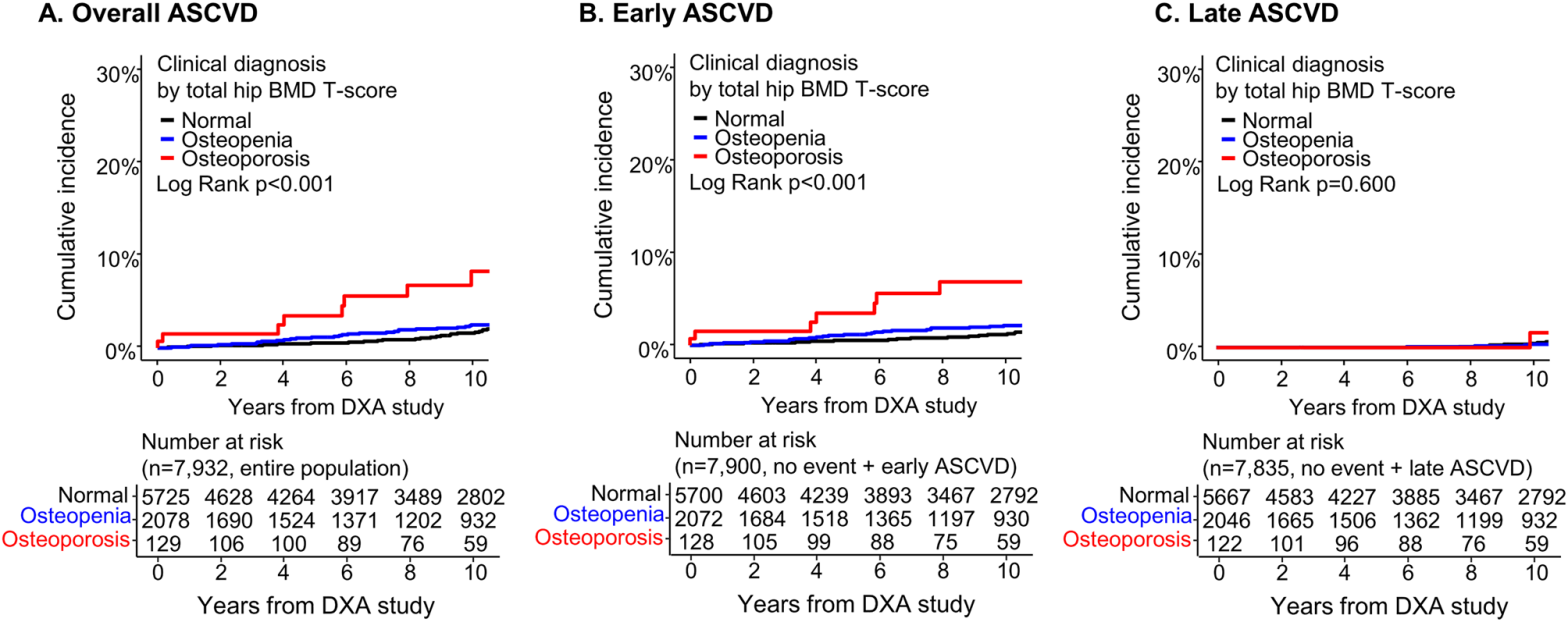
Cumulative incidence of ASCVD according to the clinical diagnosis. The figure represents cumulative incidence of overall (**A**), early (**B**), and late (**C**) ASCVD according to the clinical diagnosis of normal BMD, osteopenia, and osteoporosis. Significant differences were observed for overall and early ASCVD, but not for late ASCVD. *ASCVD* atherosclerotic cardiovascular disease, *BMD* bone mineral density, *DXA* dual-energy X-ray absorptiometry.

Univariable Cox regression analysis results for overall, early, and late ASCVD are provided in Table 2. Age, T2DM, HL, current smoking, and BMD T-score demonstrated significant associations with the overall occurrence of ASCVD. Similarly, age, T2DM, current smoking, and BMD T-score were significantly associated with early ASCVD. While age, T2DM, and HL were significant predictors of late ASCVD, the BMD T-score did not show a significant association with late ASCVD. Cumulative incidences of overall, early, and late ASCVD in patients with normal BMD, osteopenia, and osteoporosis are shown in Fig. 2.

**Table 2:** Univariate assessment of risk factors for ASCVD.

	Overall ASCVD		Early ASCVD		Late ASCVD	
	HR (95% CI)	p-value	HR (95% CI)	p-value	HR (95% CI)	p-value
Age, per 5-year increase	1.83 (1.48–2.28)	< 0.001	1.37 (1.09–1.73)	0.008	9.84 (4.33–22.34)	< 0.001
BMI, per 1 kg/m^2^ increase	1.03 (0.97–1.09)	0.326	1.03 (0.96–1.09)	0.439	1.04 (0.93–1.16)	0.521
Hypertension	0.97 (0.67–1.42)	0.883	0.94 (0.61–1.46)	0.781	1.07 (0.51–2.27)	0.854
Type 2 diabetes	4.90 (3.39–7.09)	< 0.001	4.66 (3.03–7.17)	< 0.001	5.99 (2.93–12.26)	< 0.001
Hyperlipidemia	2.00 (1.40–2.84)	< 0.001	1.58 (1.06–2.36)	0.025	5.03 (2.07–12.24)	< 0.001
Current smoking	4.05 (1.89–8.68)	< 0.001	4.76 (2.08–10.88)	< 0.001	2.22 (0.30–16.24)	0.434
Previous fracture	1.32 (0.54–3.23)	0.542	1.45 (0.53–3.95)	0.466	0.97 (0.13–7.13)	0.979
Total hip BMD, per 1-SD decrease	1.32 (1.11–1.57)	0.002	1.34 (1.10–1.65)	0.005	1.25 (0.88–1.78)	0.214
Total hip T-score, per 1-SD decrease	1.30 (1.10–1.55)	0.002	1.34 (1.10–1.64)	0.003	1.20 (0.85–1.68)	0.304
**Diagnosis of osteopenia and osteoporosis based on total hip T-score**						
Normal BMD (− 1 < T-score)	1 (reference)		1 (reference)		1 (reference)	
Osteopenia (− 2.5 < T-score ≤ − 1)	1.32 (0.90–1.93)	0.162	1.56 (1.02–2.41)	0.042	0.71 (0.29–1.74)	0.455
Osteoporosis (T-score ≤ − 2.5)	4.38 (2.12–9.05)	< 0.001	5.42 (2.48–11.88)	< 0.001	1.90 (0.26–14.05)	0.528

*ASCVD* atherosclerotic cardiovascular disease, *BMD* bone mineral density, *BMI* body mass index, *CI* confidence interval, *HR* hazard ratio, *SD* standard deviation.

**Figure 3:**
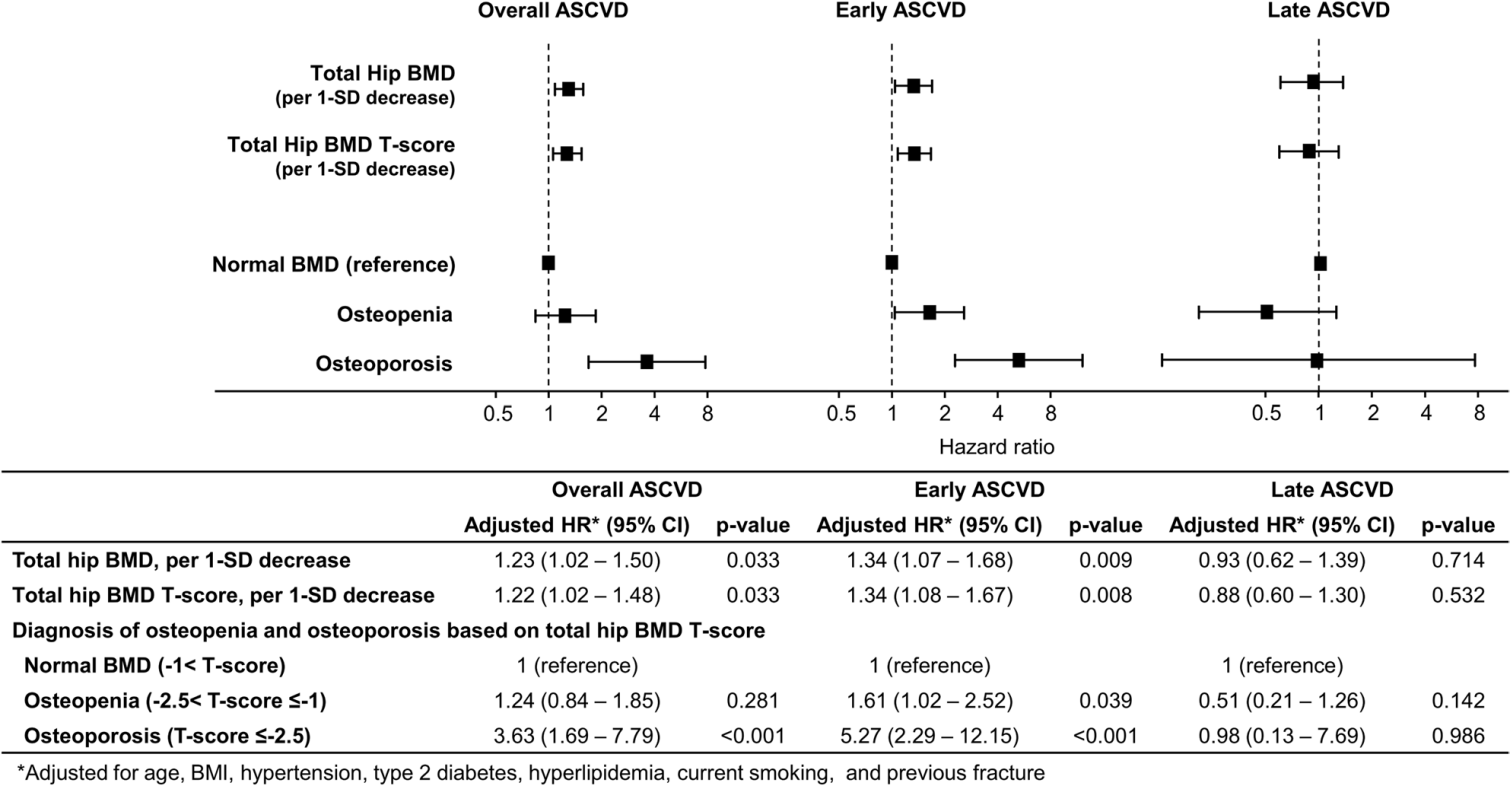
Independent predictors of ASCVD. Total hip BMD, BMD T-score, and a diagnosis of osteoporosis were independent predictors for the overall occurrence of ASCVD. The BMD and BMD T-score were independently associated with the risk of early ASCVD, but not late ASCVD. Similarly, the diagnoses of osteopenia and osteoporosis exhibited independent associations with the risk of early ASCVD, but not late ASCVD. *ASCVD* atherosclerotic cardiovascular disease, *BMD* bone mineral density, *BMI* body mass index, *CI* confidence interval, *HR* hazard ratio, *SD* standard deviation.

Multivariable Cox regression analysis results for overall, early, and late ASCVD are provided in Table 3. The BMD T-score and a diagnosis of osteoporosis demonstrated independent associations with the overall occurrence of ASCVD (adjusted HR 1.22 per 1-SD decrease, 95% confidence interval [CI] 1.02–1.48, p = 0.033; and adjusted HR 3.63, 95% CI 1.69–7.79, p < 0.001, respectively). The BMD T-score maintained an independent association with the risk of early ASCVD (adjusted HR 1.34 per 1-SD decrease, 95% CI 1.08–1.67, p = 0.008), but not with the risk of late ASCVD (adjusted HR 0.88, 95% CI 0.60–1.30, p = 0.532) (Fig. 3). Similarly, the presence of osteopenia and osteoporosis showed independent associations with early ASCVD (adjusted HR 1.61, 95% CI 1.02–2.52, p = 0.039; and adjusted HR 5.27, 95% CI 2.29–12.15, p < 0.001, respectively), but not with late ASCVD (adjusted HR 0.51, 95% CI 0.21–1.26, p = 0.142; and adjusted HR 0.98, 95% CI 0.13–7.69, p = 0.986, respectively) (Fig. 3, Table 3).

**Table 3:** Multivariate assessment of independent predictors for ASCVD.

	Overall ASCVD			Early ASCVD			Late ASCVD	
	Adjusted HR (95% CI)	p-value		Adjusted HR (95% CI)	p-value		Adjusted HR (95% CI)	p-value
**Multivariate model with total hip T-score**								
Age, per 5-year increase	1.61 (1.28–2.02)	< 0.001		1.77 (1.31–2.38)	< 0.001		9.88 (4.25–22.97)	< 0.001
BMI, per 1 kg/m^2^ increase	1.00 (0.94–1.06)	0.922		1.01 (0.94–1.07)	0.863		0.95 (0.84–1.08)	0.441
Hypertension	0.96 (0.66–1.40)	0.831		0.95 (0.61–1.48)	0.827		1.06 (0.50–2.26)	0.872
Type 2 diabetes	3.96 (2.69–5.84)	< 0.001		4.22 (2.68–6.66)	0.003		3.56 (1.70–7.45)	< 0.001
Hyperlipidemia	1.58 (1.10–2.29)	0.014		1.29 (0.85–1.95)	0.232		3.81 (1.54–9.45)	0.004
Current smoking	2.95 (1.37–6.36)	0.006		3.59 (1.56–8.27)	0.003		1.24 (0.19–9.38)	0.832
Previous fracture	1.06 (0.43–2.60)	0.903		1.23 (0.45–3.35)	0.688		0.75 (0.10–5.59)	0.782
Total hip T-score, per 1-SD decrease	1.22 (1.02–1.48)	0.033		1.34 (1.08–1.67)	0.008		0.88 (0.60–1.30)	0.532
**Multivariate model with diagnosis of osteopenia and osteoporosis based on total hip T-score**								
Age, per 5-year increase	1.64 (1.31–2.05)	< 0.001		1.78 (1.33–2.39)	< 0.001		9.96 (4.34–22.84)	< 0.001
BMI, per 1 kg/m^2^ increase	1.00 (0.94–1.06)	0.911		1.01 (0.94–1.07)	0.837		0.95 (0.84–1.08)	0.435
Hypertension	0.96 (0.66–1.40)	0.829		0.95 (0.61–1.47)	0.807		1.07 (0.51–2.27)	0.861
Type 2 diabetes	3.99 (2.71–5.89)	< 0.001		4.32 (2.74–6.81)	< 0.001		3.47 (1.66–7.27)	< 0.001
Hyperlipidemia	1.58 (1.09–2.28)	0.015		1.29 (0.85–1.95)	0.229		3.74 (1.51–9.28)	0.004
Current smoking	2.84 (1.32–6.14)	0.008		3.38 (1.46–7.81)	0.004		1.17 (0.15–8.87)	0.879
Previous fracture	1.01 (0.41–2.49)	0.988		1.17 (0.43–3.22)	0.755		0.75 (0.10–5.60)	0.776
**Diagnosis of osteopenia and osteoporosis based on total hip T-score**								
Normal BMD (− 1 < T-score)	1 (reference)			1 (reference)			1 (reference)	
Osteopenia (− 2.5 < T-score ≤ − 1)	1.24 (0.84–1.85)	0.281		1.61 (1.02–2.52)	0.039		0.51 (0.21–1.26)	0.142
Osteoporosis (T-score ≤ -2.5)	3.63 (1.69–7.79)	< 0.001		5.27 (2.29–12.15)	< 0.001		0.98 (0.13–7.69)	0.986

*ASCVD* atherosclerotic cardiovascular disease, *BMD* bone mineral density, *BMI* body mass index, *CI* confidence interval, *HR* hazard ratio, *SD* standard deviation.

### ASCVD risk stratification by BMD and conventional risk factors

**Figure 4:**
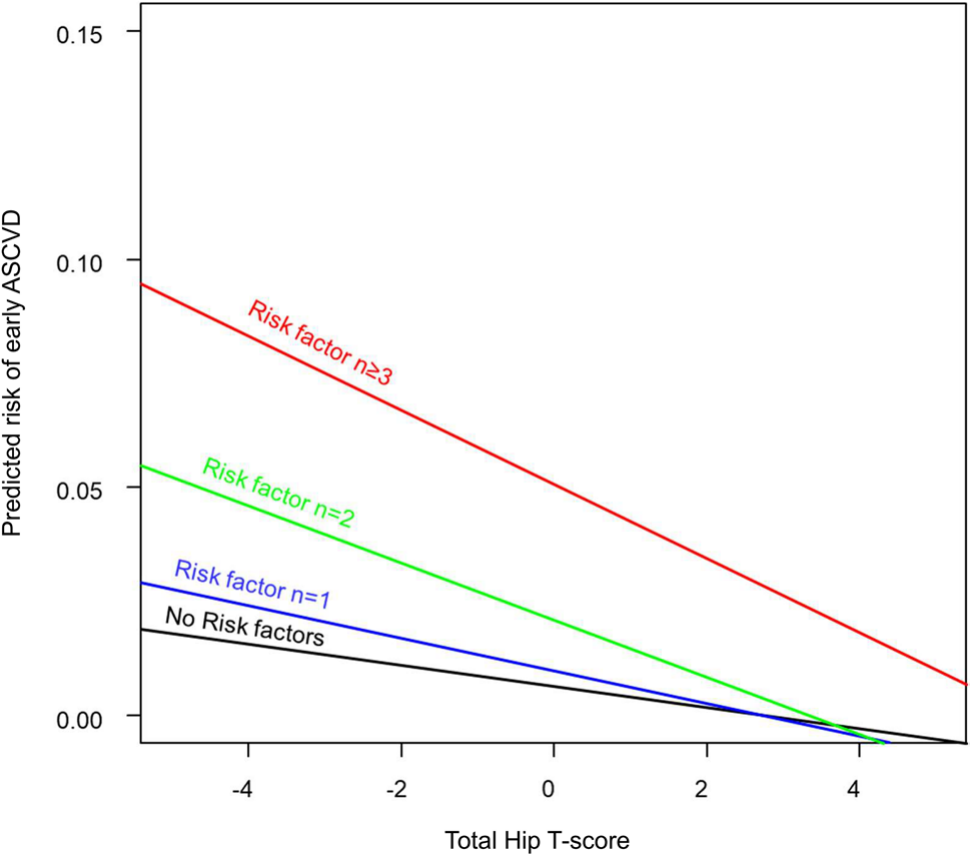
Predicted risk of early ASCVD according to BMD T-score and the number of clinical risk factors. The predicted risk of early ASCVD across the range of BMD T-scores, stratified by the number of co-existing risk factors, is shown. An inverse trend between the predicted risk and BMD T-score was consistently observed, regardless of the number of risk factors. Of note, the strength of the trend increased with a greater number of the risk factors. *ASCVD* atherosclerotic cardiovascular disease, *BMD* bone mineral density.

The predicted risk of early ASCVD demonstrated an inverse trend across the range of T-scores, with increasing ASCVD risk with a decrease in BMD T-score (Fig. 4). This trend was consistent regardless of accompanied clinical risk factors, although the strength of the trend increased with an increased number of clinical risk factors.

## Discussion

We previously reported that low BMD had independent and incremental value over age and conventional risk factors for the prediction of future ASCVD in women in a large cohort of consecutive women who underwent DXA^6^. In the present study, we additionally investigated the differential predictive value of low BMD for early and late ASCVD occurrence. Women with earlier ASCVD events, who experienced ASCVD before the age of 70 years, had lower estimated BMD T-score at the age of 65 years than women without ASCVD events. However, the estimated BMD T-score at the age of 65 years in women who experienced ASCVD at the age of 70 year or older did not significantly differ from that of women without ASCVD events. Furthermore, lower BMD was significantly associated with the occurrence of early ASCVD even after adjusting for age and clinical risk factors. In contrast, low BMD did not exhibit a significant association with late ASCVD. A significant association between low BMD and early ASCVD was consistently observed, regardless of the number of accompanied clinical risk factors; however, the strength of the trend increased with a greater number of risk factors.

Previous epidemiologic studies have reported a high prevalence of ASCVD in patients with low BMD and vice versa, suggesting a potential link between these two disease categories^3–5^. Although further clarification is needed, several pathophysiologic mechanisms have been proposed to underlie both ASCVD and osteoporosis, supporting a biologic association^1^. However, these data are counterbalanced by conflicting evidence demonstrating that the potential association between ASCVD and osteoporosis is largely an age-dependent process^15,16^, given the increased rate of incidence of both diseases among the elderly population^1,2,7^. Previous longitudinal studies also showed conflicting results. In our previous longitudinal study, which included 12,681 women who underwent DXA for osteoporosis screening, we found that low BMD was an independent predictor for ASCVD after adjustment for age and clinical risk factors^6^. Furthermore, the addition of BMD, or the clinical diagnosis of osteoporosis, to clinical risk factors significantly improved the discrimination for ASCVD. In contrast, several studies suggested that low BMD is not an independent predictor over age and clinical risk factors for cardiovascular disease in women^11,17,18^. Interestingly, the study population was relatively older in these studies (mean age 73–76 years) than in our previous study (mean age 63 years)^6^. Although we could not directly compare the age at the time of ASCVD between these conflicting studies, the difference in overall mean age provides a clue regarding the differential impact of low BMD on earlier and later ASCVD occurrence.

Several potential mechanisms may explain the link between low BMD and an earlier occurrence of ASCVD. Mishra et al. conducted a whole genome-wide association study comprising 1032 subjects aged 30–45 years, and found co-expressed genes shared by BMD impairment and carotid intima-media thickness^19^. Additionally, accelerated atherosclerosis and bone loss have been observed in patients with inflammatory rheumatic diseases, indicating the relevance of the contribution of inflammatory conditions to earlier cardiovascular disease in patients with osteoporosis^20^. Hormonal and metabolic alterations that occur during the menopausal transition in women may also expedite the rate of atherosclerosis and bone loss^21,22^. Further studies are required to fully elucidate the underlying biologic relationship between low BMD and the early onset of ASCVD.

Despite clinical observations regarding a potential association between atherosclerosis and osteoporosis, and suggested mechanisms, it remained unclear whether the prognostic value of low BMD for the occurrence of ASCVD differed according to the age on ASCVD onset. To the best of our knowledge, the present study firstly demonstrated that the prognostic value of low BMD was limited to women who experienced early ASCVD, but not late ASCVD. The differential predictive value of low BMD for only women who experienced ASCVD at relatively younger ages (i.e., women who might be at low risk based on the age factor per se), could further support a relationship between osteoporosis and ASCVD beyond aging.

One could argue that an earlier manifestation of ASCVD results from an acceleration of the disease course driven by coexisting conventional risk factors^7^. However, in the present study, the prevalences of clinical risk factors were largely comparable between the early and late ASCVD groups. In addition, although the predicted risk for early ASCVD increased with a greater number of accompanied clinical risk factors, a significant association between low BMD and early ASCVD was consistently observed regardless of the number of concomitant risk factors. Interestingly, the strength of the trend between early ASCVD and low BMD increased with an increased number of clinical risk factors. These results may suggest the clinical benefit of BMD as a sensitive indicator for predicting early ASCVD, even in women with higher comorbidity burden. Additionally, given the non-invasiveness, low risk of radiation exposure, and wide availability of DXA scans used in daily practice, the assessment of BMD can be a cost-effective method for the risk stratification of early ASCVD.

To the best of our knowledge, the current study is the first to evaluate the differential prognostic value of low BMD for ASCVD according to the event period. The strength of this study comes from its use of a large study population comprising consecutive women; thus, we believe that our study reflects real-world clinical settings and provides relevant findings. However, our results need to be interpreted with consideration of the following limitations. Due to the retrospective study design, unmeasured potential confounders may exist, affecting the association between low BMD and ASCVD, despite multivariable adjustment for age and clinical risk factors. For instance, we could not precisely assess physical activity levels, which can affect both BMD and ASCVD risk. Prospective studies that include quantitative and qualitative assessment of these potential confounders are required. Furthermore, the study participants consisted of relative younger subjects, with a lower burden of cardiovascular risk factors and small event numbers, thus it may have limited our results, rendering the analyses underpowered for a firm conclusion. Additionally, the current study results should be interpreted with caution, especially when extrapolating in older age patients with higher risk features. Further studies in a cohort with a higher event rate would be helpful to evaluate whether the current study results can be extrapolated to those at higher risk. Nevertheless, the clinical implication of the current study remains clear, as we demonstrated the clinical value of BMD assessment for predicting early ASCVD in a relatively healthy population, where there is a shortage of risk assessment tools for early cardiovascular events. The data for chronic inflammatory disease and their relevant parameters, which could be a potential mediator underlying both bone and vasculature system, were not available in the current study. Further studies would be of clinical value, evaluating how the inflammatory process affects the observed association between low BMD and ASCVD. Finally, since the study population was derived from a cohort of consecutive women from a single tertiary center in South Korea; the definition for early and late ASCVD using the age of 70 years as a cutoff was also derived based on the Korean nationwide study, reporting the peak age group of ASCVD as 70–79 years^7^. Therefore, our results are limited in their application to other ethnic groups, with different clinical characteristics, and men.

In conclusion, low BMD is an independent predictor, over age and clinical risk factors, for early ASCVD in women. Thus, the assessment of BMD in women can provide prognostic benefit for risk stratification for earlier ASCVD occurrence. Further investigations are required to elucidate the underlying biological links between low BMD and the early onset of ASCVD.

## Supplementary Information

Supplementary Information.

## Data Availability

The datasets analyzed during the current study are not publicly available due to potentially sensitive patient information but are available from the corresponding author on reasonable request.

## Abbreviations

ASCVD: Atherosclerotic cardiovascular disease
BMD: Bone mineral density
BMI: Body mass index
CI: Confidence interval
DXA: Dual-energy X-ray absorptiometry
HL: Hyperlipidemia
HR: Hazard ratio
HTN: Hypertension
IQR: Interquartile range
MI: Myocardial infarction
SD: Standard deviation
T2DM: Type 2 diabetes mellitus
