# Supplementary material for: Risk of earlier atherosclerotic cardiovascular disease in women with low bone mineral density
Source: Sci Rep. 2022 Sep 26;12:15996. doi: 10.1038/s41598-022-19801-5 (PMC9512928; doi:10.1038/s41598-022-19801-5)

Risk of earlier atherosclerotic cardiovascular disease in women with low bone mineral density

Jiesuck Park, Kyoung Min Kim, Yeonyee E. Yoon, In-Chang Hwang, Goo-Yeong Cho

Supplementary Material

Supplementary Table S1 online. Baseline characteristics of the entire study population and according to ASCVD event

Supplementary Fig. S1 online. Distribution of estimated BMD T-scores at 65 years of age

Supplementary Table S1 online. Baseline characteristics of the entire study population and according to ASCVD event

	Entire study population (n=7,932)	No event (n= 7,803)	Overall ASCVD (n= 129)	p-value vs. no event
**Age, years**	58 (54 – 62)	58 (54 – 62)	61 (58 – 63)	<0.001
**BMI, kg/m^2**	23.6 (21.8 – 25.6)	23.6 (21.8 – 25.6)	24.2 (22.2 – 25.9)	0.221
**Hypertension**	2,383 (30.0)	2,345 (30.1)	38 (29.5)	0.961
**Type 2 diabetes**	684 (8.6)	642 (8.2)	42 (32.6)	<0.001
**Hyperlipidemia**	2,871 (36.2)	2,793 (35.8)	78 (60.5)	<0.001
**Current smoking**	94 (1.2)	87 (1.1)	7 (5.4)	<0.001
**Previous fracture**	182 (2.3)	177 (2.3)	5 (3.9)	0.361
**Total hip T-score**	-0.4 (-1.0 – 0.3)	-0.4 (-1.0 – 0.3)	-0.6 (-1.2 – 0.1)	0.009
**Diagnosis of osteopenia and osteoporosis based on total hip T-score**				
**Normal BMD (-1< T-score)**	5,725 (72.2)	5,642 (72.3)	83 (64.3)	<0.001
**Osteopenia (-2.5< T-score ≤-1)**	2078 (26.2)	2,040 (26.1)	38 (29.5)	
**Osteoporosis (T-score ≤-2.5)**	129 (1.6)	121 (1.6)	8 (6.2)	

Data are presented as the median (interquartile range) for continuous variables and number (percentage) for categorical variables
Abbreviations: ASCVD, atherosclerotic cardiovascular disease; BMD, bone mineral density; BMI, body mass index

Supplementary Figure 1. Distribution of estimated BMD T-scores at 65 years of age

Abbreviation: ASCVD, atherosclerotic cardiovascular disease

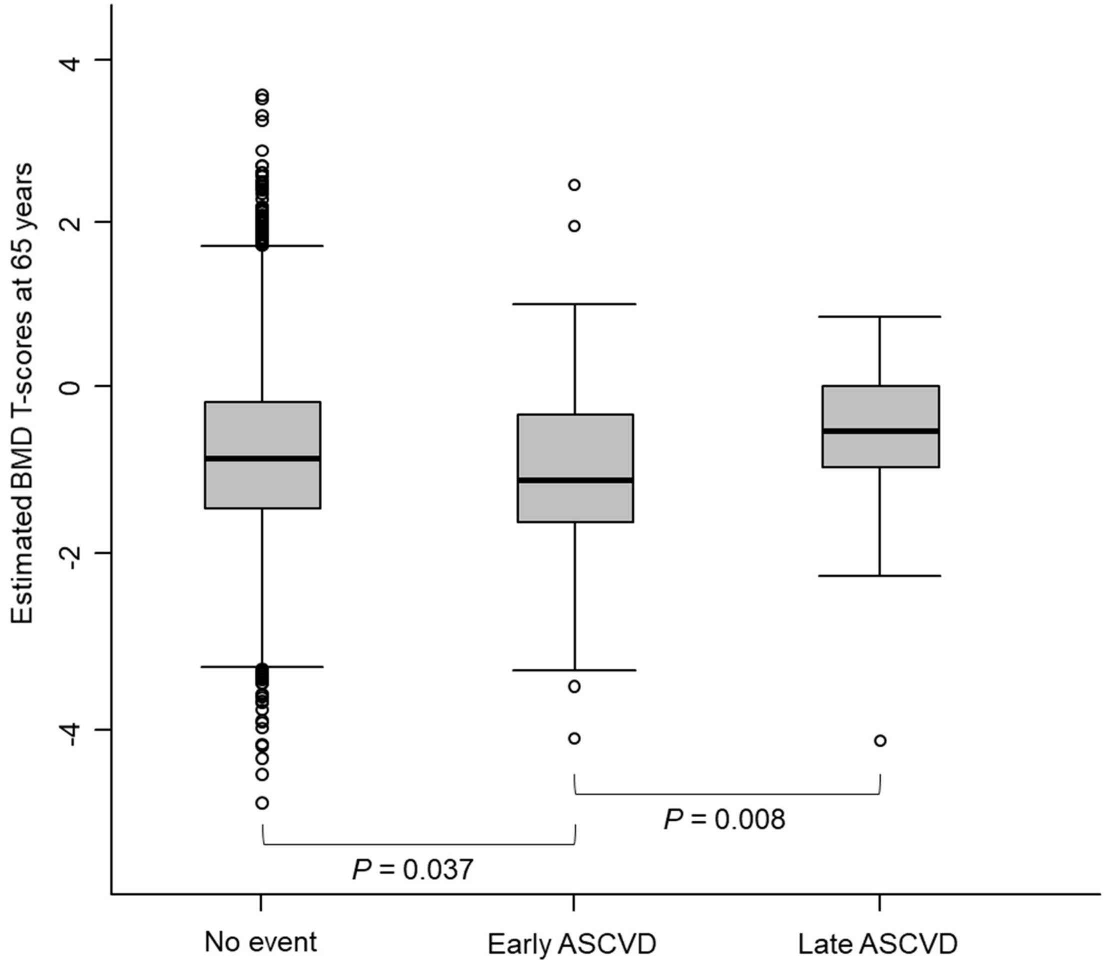

Supplement: Supplementary file 1 — Supplementary Information. [file 41598_2022_19801_MOESM1_ESM.pdf]
